# Parental knowledge of paediatric vaccination

**DOI:** 10.1186/1471-2458-9-154

**Published:** 2009-05-27

**Authors:** Eva Borràs, Àngela Domínguez, Miriam Fuentes, Joan Batalla, Neus Cardeñosa, Antoni Plasencia

**Affiliations:** 1 CIBER Epidemiología y Salud Pública (CIBERESP), Barcelona, Spain; 2 Directorate of Public Health, Department of Health, Generalitat of Catalonia, Roc Boronat 81-95, 08005 Barcelona, Spain; 3 Department of Public Health, University of Barcelona, Barcelona, Spain

## Abstract

**Background:**

Although routine vaccination is a major tool in the primary prevention of some infectious diseases, there is some reluctance in a proportion of the population. Negative parental perceptions of vaccination are an important barrier to paediatric vaccination. The aim of this study was to investigate parental knowledge of paediatric vaccines and vaccination in Catalonia.

**Methods:**

A retrospective, cross-sectional study was carried out in children aged < 3 years recruited by random sampling from municipal districts of all health regions of Catalonia. The total sample was 630 children. Parents completed a standard questionnaire for each child, which included vaccination coverage and knowledge about vaccination. The level of knowledge of vaccination was scored according to parental answers.

**Results:**

An association was observed between greater vaccination coverage of the 4:4:4:3:1 schedule (defined as: 4 DTPa/w doses, 4 Hib doses, 4 OPV doses, 3 MenC doses and 1 MMR dose) and maternal age >30 years (OR: 2.30; 95% CI: 1.20–4.43) and with a knowledge of vaccination score greater than the mean (OR: 0.45; 95% CI: 0.28–0.72). The score increased with maternal educational level and in parents of vaccinated children.

A total of 20.47% of parents stated that vaccines could have undesirable consequences for their children. Of these, 23.26% had no specific information and 17.83% stated that vaccines can cause adverse reactions and the same percentage stated that vaccines cause allergies and asthma.

**Conclusion:**

Higher vaccination coverage is associated with older maternal age and greater knowledge of vaccination.

Vaccination coverage could be raised by improving information on vaccines and vaccination.

## Background

Although routine vaccination is a major tool in the primary prevention of some infectious diseases, a proportion of the population are reluctant to receive vaccines. Rumours of a possible association between hepatitis B vaccination and multiple sclerosis and between the mumps, measles and rubella (MMR) vaccine and autism have affected vaccination coverages in some countries, especially those with antivaccination movements [1-3]. Spanish studies show positive parental perceptions to vaccination [4,5]. However, this may have been modified by the impact of news reports and publications that influence attitudes to vaccines and increase rejection of vaccination, as has happened in other countries [6-8]. When the incidence of vaccine-preventable diseases falls, awareness of the risk of possible adverse events increases, leading to reduced vaccination coverages [9]. Negative parental perceptions of vaccination have been identified as an important barrier to paediatric vaccination [10]. Therefore, it is important to understand which variables influence parental decisions to vaccinate their children or not. Some studies [11-18] show that healthcare providers have a positive influence on parental decisions to vaccinate their children, including parents who believe that vaccinations are unsafe [19].

Factors that increase acceptance include the desire to prevent disease, helping the community by means of herd immunity and doing what other people do. Possible factors reducing acceptance are the fear of harming their child, the belief that their child is not at risk because other children are vaccinated, the perception that they are able to control the child's susceptibility and the outcome of the disease, the opinion that natural immunity after suffering the disease is better than vaccination, doubts about the reliability of information on vaccination, and mistrust of vaccine safety and risk [10,12,14,20].

The objective of this study was to investigate the influence of parental knowledge of vaccines and vaccination in Catalonia, a region in the northeast of Spain with seven million inhabitants.

## Methods

A retrospective cross-sectional study was carried out. Children born in October 2001 in Catalonia were recruited by random sampling of municipal districts of all Catalan health regions. Participants were randomly selected from municipal electoral registers.

The sample size was calculated with a precision of 0.05 and an expected probability of routine vaccination coverage of 0.97. Thus, the sample size required was 45 children from rural and 45 from urban habitats for each of the seven health regions except for Barcelona city, giving a total sample of 630 children.

Vaccines studied were those included in the current routine vaccination schedule recommended for Catalonia: diphtheria, tetanus and whole-cell or acellular pertussis (DTPa/w), trivalent oral polio (OPV), *Haemophilus influenzae *type b (Hib), conjugated meningococcal C (MenC) and measles, mumps and rubella (MMR) vaccines. The 3:3:3:3:1 vaccination schedule was defined as having received 3 DTPa/w doses, 3 Hib doses, 3 OPV doses, 3 MenC doses and 1 MMR dose. The 4:4:4:3:1 schedule was defined as: 4 DTPa/w doses, 4 Hib doses, 4 OPV doses, 3 MenC doses and 1 MMR dose. All children who received the 4:4:4:3:1 series had also received the 3:3:3:3:1 series.

A telephone survey of parents of selected children was carried out between October 2003 and September 2004. Only 12 (1.90%) families refused to participate and 25 (3.97%) families could not be located. These families were replaced by reserves until the number of 630 was reached.

Parents completed a standard questionnaire [5,21,22] for each child, which included vaccinations received and knowledge of vaccines and vaccination. The questionnaire collected data on: person answering the questionnaire, parent's date of birth, parent's educational level (a high level was classified as professional training level II, high school graduation, baccalaureate, or above) and occupational status, parental knowledge of vaccines and vaccination and type and age of vaccine administration.

Parents were requested to send a photocopy of the child's vaccination card to validate the data provided.

No participant was excluded due to language difficulties. When necessary a new appointment was made in which a family member could act as a translator.

The study was approved by the Bioethics Committee of the Department of Health, Generalitat of Catalonia.

### Statistical analysis

Parent's knowledge of vaccination was scored by counting the number of correct answers to nine questions (maximum score 9 points). The questions and answers considered correct are shown in Table 1.

**Table 1:** Questions and answers considered most-correct on knowledge about vaccination.

**Questions of score of knowledge about vaccination**	**Most-correct answers**
*Age at initiation of routine schedule*	*2–3 months*
*Are vaccines necessary?*	*Yes*
*Do you think vaccine-preventable diseases are potentially?*	*Severe/very severe*
*Would you vaccinate in summer?*	*Yes*
*Would you vaccinate with a cold?*	*Yes*
*Would you vaccinate with fever (>38°C)?*	*No*
*Are vaccines harmful?*	*No*
*What may happen to a pregnant woman with rubella?*	*Foetal malformations*
*Measles*	*May produce complications*.

Mothers were divided into two groups (≤ 30 years and >30) in accordance with the median age of mothers in Catalonia in 2001 [23].

The statistical analysis was carried out using the SPSS v15.0.1 for Windows statistical package; copyright (c) SPSS Inc., 1989–2006.

A value of p < 0.05 was considered as statistically significant. The prevalences and their 95% confidence intervals (CI) were calculated by the exact binomial method and the differences between proportions were analysed using the Chi^2 ^test.

Associations between the coverage and the studied variable were calculated using the odds ratio (OR) and their 95% CI. The mean, median and standard deviation (SD) of the parents' scores were calculated. Comparisons of the quantitative variables were made using the Kruscal-Wallis non parametric test as normality was not fulfilled according to Kolmogorov and Shapiro-Wilk [24-26].

## Results

The telephone questionnaire was answered by the mother in 87.62% (552/630) of cases, with a mean age of 34.08 ± 4.59 years and by the father in 10.80% (68/630) of cases, with a mean age of 37.35 ± 5.46 years. In the remaining 10 cases, the questionnaire was answered by grandparents. Data were obtained by the parent reading directly from the vaccination card. The vaccination card was sent for study by 46.88% (294/627) of parents (in three cases the parents stated they had no vaccine card as they were opposed to vaccines) and the information coincided in 100% of cases.

Maternal age was recorded in 310 mothers and an association was observed between maternal age >30 years and higher vaccination coverage for all the vaccination schedules studied (Table 2).

**Table 2:** Coverage of vaccination schedules according to maternal age.

Vaccination	Maternal age					
Schedules	>30 years (n = 241)			≤ 30 years (n = 69)		
	
	n	%	95% CI	n	%	95% CI

3:3:3:3:1*^&^	227	94.19	91.03–97.35	60	86.96	78.28–95.63

4:4:4:3:1**^&&^	209	86.72	82.23–91.21	51	73.91	62.83–85.0

*3:3:3:3:1 series was defined as 3 DTPa/w doses, 3 Hib doses, 3 OPV doses, 3 MenC doses and 1 MMR dose.

**4:4:4:3:1 series was defined as: 4 DTPa/w doses, 4 Hib doses, 4 OPV doses, 3 MenC doses and 1 MMR dose.

^&^OR: 2.43 (1.00–5.89); p: 0.04

^&&^OR: 2.30 (1.20–4.43); p: 0.01

No significant association was observed between vaccination coverage and the educational level (high or low) of the parent (father or mother) who answered the questionnaire.

Neither was an observation observed between the mother's occupational status and vaccination coverage. For the 4:4:4:3:1 schedule, coverage in working mothers was 87.32% compared with 84.73% in non-working mothers (OR:1.24; 95% CI: 0.77–2.01), and for the 3:3:3:3:1 schedule the respective values were 95% and 93.10% (OR: 1.44; 95% CI: 0.71–2.92).

Of the parents interviewed, 68.9% thought that vaccination should be obligatory, 20.3% that it should remain voluntary and 10.8% offered no opinion.

A small percentage of children had not received one or more of the vaccines studied: 0.63% (95% CI: 0.17–1.62) had received no dose of DTP, 0.47% (95% CI: 0.098–1.38) no dose of OPV, 1.11% (95% CI:0.21–2.01) no dose of Hib and 0.79% (95% CI: 0.26–1.84) no dose of MenC. The highest percentage of unvaccinated children was observed for the MMR vaccine (1.58%; 95% CI: 0.53–2.64). Three children, whose parents were opposed to vaccination, had not received any vaccine (0.48%; 95% CI: 0.09–1.40). These three families stated that vaccination should remain voluntary.

Vaccines were perceived as necessary by 89% of parents interviewed and 60% stated that vaccine-preventable diseases were severe or very severe. Better compliance with the 4:4:4:3:1 schedule was observed among these respondents.

Table 3 shows specific parental answers to questions on knowledge of vaccines and vaccination according to percentages of vaccinated and non-vaccinated children for the 3:3:3:3:1 and 4:4:4:3:1 schedules.

**Table 3:** Specific answers to questions on knowledge of vaccines and vaccination according to percentages of vaccinated and non-vaccinated children for the 3:3:3:3:1 and 4:4:4:3:1 schedules.

		**3:3:3:3:1 schedule**		**4:4:4:3:1 schedule**	
		
**Questions**	**n (630)**	**Vac %** **(95%CI)**	**Non-vac %** **(95%CI)**	**Vac %** **(95%CI)**	**Non-vac %** **(95%CI)**
Initiation of routine schedule at 2–3 months	381	94.23 (91.75–96.70)	5.77 (3.30–8.25)	87.14 (83.65–90.63)	12.86 (9.37–16.35)
Are vaccines necessary? Yes	559	95.53* (93.72–97.33)	4.47* (2.67–6.27)	87.66** (84.84–90.47)	12.34** (9.53–15.16)
Do you think vaccine-preventable diseases are potentially? Severe/very severe	376	95.48 (93.25–97.71)	4.52 (2.29–6.75)	88.03 (84.62–91.45)	11.97 (8.55–15.38)
Would you vaccinate in summer? Yes	410	94.88 (92.62–97.13)	5.12 (2.86–7.38)	87.07 (83.70–90.44)	12.93 (9.56–16.30)
Would you vaccinate with a cold? Yes	56	98.21 (90.45–99.95)	1.79 (0.04–9.55)	89.28 (80.29–98.28)	10.71 (1.72–19.71)
Would you vaccinate with fever (>38°C)? No	554	94.77 (92.82–96.71)	5.23 (3.29–7.18)	86.82 (83.92–89.73)	13.18 (10.27–16.08)
Are vaccines harmful? No	400	96.75^&^ (94.89–98.61)	3.25^&^ (1.39–5.11)	88.0 (84.69–91.31)	12.0 (8.69–15.31)
What may happen to a pregnant woman with rubella? Foetal malformations	430	96.05^&&^ (94.09–98.01)	3.95^&&^ (1.99–5.91)	89.77^$^ (86.79–92.75)	10.23^$^ (7.25–13.21)
Measles: Can produce complications? Yes	259	94.98 (92.13–97.83)	5.02 (2.17–7.87)	88.42 (84.33–92.51)	11.58 (7.49–15.67)

* OR: 4.34 (2.07–9.09); p < 0.0001; ** OR: 2.23 (1.22–4.075); p: 0.007; ^&^OR: 3.47 (1.73–6.95); p: 0.0002;

^&&^OR: 2.70 (1.38–5.27); P: 0.003; ^$ ^OR: 2.33 (1.47–3.70); P: 0.0002

Believing that vaccines are necessary and that becoming infected with rubella can result congenital malformations, was associated with higher coverage of the two schedules studied (Table 3), while believing vaccines do no harm was only associated with the 3:3:3:3:1 schedule.

A higher score was observed in parents of vaccinated children compared with those of non vaccinated children. Better compliance with the vaccination schedule was found in parents with a higher score; the score of parents of children not vaccinated with the 4:4:4:3:1 schedule was higher than that of those not vaccinated with the 3:3:3:3:1 schedule (Table 4).

**Table 4:** Descriptive statistics of parental knowledge of vaccination scores according to the vaccination schedule.

**Vaccination schedule**	**n_i_**	**Mean**	**SD**	**Median**	**Min-Max**	**p**
3:3:3:3:1*						
Yes	593	5.51	1.56	6.00	0–9	<0.001
No	37	4.19	2.08	4.00	0–8	
4:4:4:3:1**						
Yes	544	5.53	1.56	6.00	9-9	0.001
No	86	4.81	1.88	5.00	0–8	

**Total**	630	5.43	1.63	6.00	0–9	

*3:3:3:3:1 series was defined as: 3 DTPa/w doses, 3 Hib doses, 3 OPV doses, 3 MenC doses and 1 MMR dose.

**4:4:4:3:1 series was defined as: 4 DTPa/w doses, 4 Hib doses, 4 OPV doses, 3 MenC doses and 1 MMR dose.

Vaccination coverage was lower in children of parents who obtained scores below the mean (5.43 points) for both schedules studied. The OR was 0.39 (95% CI: 0.19–0.81; p = 0.01) for the 3:3:3:3:1 schedule and 0.45 (0.28–0.72; p < 0.001) for the 4:4:4:3:1 schedule.

Scores increased with maternal educational level. The mean score in mothers with a university education (n = 166) was 6.01 (SD: 1.55; median: 6). The differences were statistically significant ( = 14.267; p = 0.003) and with a decreasing linear trend in parallel to the level of education (Z_o _= -3.267; p = 0.001).

The 35 parents who had doubts about vaccination expressed different reasons: 31.43% (95% CI: 14.62–48.24) stated they were due to lack of information, 31.43% (95% CI: 14.62–48.24) to distrust of vaccine safety and risks, 25.71% (95% CI: 9.80–41.62) believed in natural therapies, 8.60% (95% CI: 1.80–23.06) followed the advice of friends and 2.86% (95% CI: 0.07–14.92) believed that vaccines were commercially driven. Of the doubting parents, 80% vaccinated their children with the 3:3:3:3:1 schedule versus 94.95% of parents without doubts (OR: 0.21; 95% CI: 0.085–0.52; p < 0.001); and 65.71% completed the 4:4:4:3:1 schedule versus 87.56% (OR: 0.27; 95% CI: 0.13–0.57; p < 0.001).

A total of 20.47% (95% CI: 17.25–23.71) of parents stated that vaccines could have undesirable consequences for their children. Of these, 23.26% had no specific information (Table 5), 17.83% stated that vaccines can cause adverse reactions and the same percentage stated that vaccines caused allergies and asthma. Children of parents who considered vaccines not to be harmful received the 3:3:3:3:1 schedule (95.60% vs 88.37%; OR: 0.35, 95% CI: 0.17–0.69; p = 0.002) and the 4:4:4:3:1 schedule (87.42% vs 82.17%; OR: 0.66; 95% CI: 0.39–1.11; NS) more often than children of parents with doubts.

**Table 5:** Main reasons expressed by parents for negative answers to vaccination (n = 129).

**Causes**	**n**	**% (95% CI)**
Non-specific harm.	30	23.26 (15.58–30.93)
Specific harm:	99	76.74 (69.06–84.42)
Adverse reactions	23	17.83 (10.84–24.82)
Causes allergies	23	17.83 (10.84–24.82)
Risk-benefit ratio	13	10.08 (4.49–15.66)
Causes the disease vaccinated for	9	6.97 (2.19–11.76)
Bad practise of health providers	8	6.20 (1.65–10.75)
Protective attitude to children	7	5.42 (1.13–9.72)
Risk of causing autism/neurological conditions	7	5.42 (1.13–9.72)
Alters children's immunologic system	6	4.65 (0.63–8.67)
Causes disease and death	3	2.32 (0.48–6.64)

## Discussion

The importance of maternal age and educational level for vaccination coverage has been shown in various reports [6,27,28]. Our results show that mothers aged ≥ 30 years vaccinated their children more. This may be due, in part, to older mothers being influenced more by memories of the benefits of vaccination and less by current controversies. Whether the mother worked or not had no effect on vaccination coverage.

Unlike studies carried out in other countries [2,12,29,30], we found that only three children (0.48%) had received no vaccine. In addition, the percentage of children who had not received vaccination against some of the vaccine-preventable diseases included in the routine schedule was lower than that observed in other studies. These low proportions may be due to the relative weakness of antivaccination movements in Spain, with 98% of parents studied considering that vaccination is necessary.

The highest percentage of unvaccinated children corresponded to the MMR vaccine (1.58%). Non vaccination with the MMR vaccination was observed in 57% of children aged 18 months in the United Kingdom [31]. In Switzerland [32], 21.34% of children were not vaccinated against rubella in 1998, and 77.52% in Italy in 1997 [33]. In Edmonton (Canada) [34], 7% of children had received no dose of the MMR vaccine in 2002. The proliferation of negative publicity about vaccines in the mass media, especially the Internet, questioning the benefits of vaccination and leading to increased belief in natural or alternative therapies may explain these higher proportions of unvaccinated children [35].

The decreasing linear trend of the level of knowledge and opinions on vaccines with the level of maternal education (p = 0.001) observed in this study has also been described in Italy [33]. In contrast, a Spanish study [36] using data from the National Survey of Health 1993–2003 found that parents with higher educational levels had less knowledge of vaccines included in the routine schedule.

We found that children of mothers with higher levels of education had higher coverages; the low number of participants included in this group may explain why the differences were not statistically significant. Studies in the United States [27,28] and Turkey [37] found that parents with higher educational levels are less worried about vaccine safety and have greater confidence in physicians and that their children receive more vaccinations [11]. In contrast, in Switzerland [32] and Germany [38], children of university-educated parents had less probability of being vaccinated than children of parents with lower educational levels.

Although only 60% of parents perceived vaccine-preventable diseases as severe or very severe, 70% stated that vaccination should be obligatory, a lower percentage than that found in another Spanish study (98.94%) [5]. The reduction in the perceived severity of vaccine-preventable diseases may be explained by the fact that cases are increasingly rare, and this may provoke debate on the need to vaccinate. In addition, many parents felt that they did not have sufficient information or that the information supplied was not clear or correct. Although parents had doubts and thought that vaccines could be harmful, a high percentage of these parents had their children vaccinated. This emphasizes the importance of health professionals providing adequate information to parents in order to avoid an increase in negative attitudes to vaccination.

The main limitation of the study is that families who agreed to participate were probably more receptive to preventive actions than the general population, although the percentage of families who refused to participate was very low (1.90%) and only 25 families could not be located.

We consider that the possible information bias was minimal, as parents were not asked to remember whether their children had been vaccinated, but were asked by the interviewer to read the data contained in the child's vaccination card directly and chronologically. Vaccination data were later verified using the vaccination card.

The cross-sectional design may be another limitation, as the high levels of vaccination coverage in Catalonia and generally positive attitudes to the public health service could have influenced changes in attitudes and knowledge (reverse causation). Therefore, no causal inferences can be drawn from our results.

## Conclusion

The results of our study reinforce the importance of the level of parental knowledge of vaccines and vaccination. Trust between paediatricians and mothers, with clear, concise information provided in a language that parents can understand and assimilate is essential. Therefore, physicians should be educated and trained to counter negative attitudes to vaccination. Health authorities should make further efforts to promote the advantages of vaccination and underline the disadvantages of non or late paediatric immunization.

## Abbreviations

CI: confidence interval; DTPa/w: diphtheria, tetanus and whole-cell or acellular pertussis; Hib: *Haemophilus influenzae *type b; MenC: conjugated meningococcal C; MMR: measles, mumps and rubella; OPV: trivalent oral polio; OR: odds-ratio; SD: standard deviation.

## Competing interests

The authors declare that they have no competing interests.

## Authors' contributions

AD and EB designed the study and drafted the manuscript. EB participated in data collection and analyses and AD supervised the statistical analysis. JB participated in the design of the sample selection. MF, NC, and AP drafted the manuscript. All authors read and approved the final manuscript.

## Pre-publication history

The pre-publication history for this paper can be accessed here:


